# Determination of Mandibular Position and Mouth Opening in Healthy Patients and Patients with Articular and/or Muscular Pathology: A Pilot Study with 3D Electromagnetic Articulography and Surface Electromyography

**DOI:** 10.3390/jcm12144822

**Published:** 2023-07-21

**Authors:** Camila Cerda, María Florencia Lezcano, Franco Marinelli, Josefa Alarcón, Ramón Fuentes

**Affiliations:** 1 Master Program in Dental Science, Dental School, Universidad de La Frontera, Temuco 4780000, Chile; camilaacerdat@gmail.com; 2 Cybernetics Laboratory, Department of Bioengineering, Facultad de Ingeniería, Universidad Nacional de Entre Ríos, Oro Verde 3100, Argentina; lezcano.f@gmail.com; 3 Research Centre in Dental Sciences (CICO-UFRO), Dental School, Facultad de Odontología, Universidad de La Frontera, Temuco 4780000, Chile; marinelli.fran@gmail.com (F.M.); josefa.alarcon@ufrontera.cl (J.A.); 4 Doctoral Program in Morphological Sciences, Faculty of Medicine, Universidad de La Frontera, Temuco 4780000, Chile; 5 Department of Integral Adults Dentistry, Dental School, Facultad de Odontología, Universidad de La Frontera, Temuco 4780000, Chile

**Keywords:** EMA, temporomandibular joint, joint disorders, muscle disorders

## Abstract

Temporomandibular disorders (TMDs) are a group of pathologies that affect the temporomandibular joint and its related structures, producing intracapsular and muscular pathologies. The aim of this study is to describe, by electromagnetic articulography (EMA) and simultaneous electromyography (sEMG), the mandibular postural position and mouth opening in healthy patients and with articular and/or muscular pathology. Materials and methods: A pilot study was conducted with a sample of sixteen participants aged 18 years or older who attended the TMDs and Orofacial Pain Polyclinic of the University of La Frontera due to TMDs. The physiological inoculation space was evaluated from the mandibular postural position (MPP) with swallowing command and without command, in both healthy patients and patients with articular, muscular, and mixed TMDs, measured simultaneously with EMA and sEMG. An angular measurement of the oral opening was also performed with the data obtained. Results: The physiological inoculation space was obtained from the determination of the MPP through the procedures with swallowing command and without command, and different mouth opening degrees were evaluated. Conclusions: Simultaneous position and sEMG records can be produced from EMA, and different characterization variables such as the vertical distance, Euclidean distance, and angle can be obtained.

## 1. Introduction

In odontology, the stomatognathic system is evaluated for academic, research, and clinical purposes with diagnostic and therapeutic objectives. It includes mandibular movements and positions. Among the usual evaluations, there are the mouth opening, the maximum intercuspation position, and the mandibular postural position, which can be used in the determination of the vertical dimension (VD).

The mandibular postural position (MPP) is the relation between the mandible and the maxilla when a person is seated with their eyes facing the horizon, in an orthostatic position, with the lips slightly touching; the related muscles are in a state of minimal contraction and without dental contact, leaving a free interdental space [1,2]. This was previously called the mandibular resting position, but nowadays it is known that the position is maintained by a tonic contraction against gravity, so the levator musculature is not in a resting state or in the absence of electromyographic activity, but rather there is constant contractile work. This position is the most common one because it is the beginning of all functional movements and is maintained by the ligaments, a balance mainly between the levator and depressor muscles of the mandible and the temporomandibular joint. There is also a dental contact position known as the maximum intercuspation position (MIC) [2], where there is a relation between the mandible and the maxilla through the greatest number of dental contacts; it is a stable position achieved through the contraction of the levator muscles, by sufficient electrical activity, to keep them in contact.

The evaluation of maxillomandibular relations can be conducted in a vertical, horizontal, and sagittal manner. The vertical evaluation corresponds to the vertical dimension, which is defined as the measurement of the anterior facial height determined between two arbitrarily defined points, one in the maxilla, commonly at the base of the nose, and the other in the mandible, commonly at the most prominent area of the chin [3]. The VD is relevant when complex oral rehabilitations are required, where lost or absent occlusion needs to be restored. Different types of VD are described, but the main one are occlusal VD (OVD), which corresponds to the height of the lower third of the face when the patient is in the MIC dental position, and postural VD, which is measured in the same way as OVD, but in the mandibular postural position [2,4]. Different methods are described in order to measure the vertical dimension [5,6], among which methods based on pre-extraction, cephalometric radiographs, esthetics, pre-extraction phonetics, tactile sensitivity, swallowing patterns, maximum biting force, the use of a rest position as a reference, and others are described [7]. However, there are no significant differences between them [8,9]. An important reference for the evaluation of VD is the determination of the physiological inocclusion space or interocclusal clearance, which is the distance between the MIC and MPP. DV has been evaluated in patients with temporomandibular disorders (TMDs) using different methodologies [10], and the good adaptation of the musculature in the face of variations in the vertical dimension is described [11,12].

In recent years, static and dynamic mandibular movements have been studied with different measurement tools, including cinefluorography, cineradiography, gnathic replicators, kinesiography, syronatography, and electrognathography, which are characterized by describing the results in one realm of space [13]. In 1994, studies with measurement tools in three realms of space began [14]. There are protocols for the measurement of chewing, swallowing, and borderline mandibular movements using electromagnetic articulography (EMA) (AG501 3D articulograph) [13,15,16,17], which has the advantages of reducing the margin of error of the measurements to a significant extent [1], as well as the evaluation of movements in the three axes of space: frontal, coronal, and sagittal. In addition, this system enables the evaluation of geometric and kinematic characteristics.

TMDs are a group of pathologies that affect the temporomandibular joint and its related structures [18], which correspond to the chewing, masseter, temporal, medial, and lateral pterygoid muscles. The stabilization of the temporomandibular joint (TMJ) is given by the major ligaments, which are the temporomandibular, stylomandibular, and sphenomandibular ligaments [18]. The prevalence of TMJ is described to be 15–20% of the adult population and it peaks between the ages of 20 and 40 years old [19]. TMDs are more common in women than in men, at a 4.1:1 ratio [20]. When any of these structures is affected, patients usually present clinical characteristics such as pain and limitation of the range of motion, commonly associated with joint noise. As they correspond to a different group of anatomical structures, different tools have been proposed for the diagnosis of TMDs, which have varied over time. In 1960, W. Bell proposed a classification differentiating intracapsular (articular) from extracapsular (muscular) pathologies [21]. In 1994, diagnostic criteria were created for the first time in order to improve studies in the area, called the Research Diagnostic Criteria for TMDs (RDC/TMDs) [22], being updated in 2014 and renamed as the Diagnostic Criteria for Temporomandibular Disorders (DC/TMDs), with the purpose of also being used them in clinical practice and not only in research [2].

Good adaptation of the musculature in the face of an increase in the vertical dimension is described [11,12]. However, there is no description of how it may behave in differentiated diagnoses with valid tools for the diagnosis of temporomandibular disorders. Therefore, the aim of this pilot study is to describe the determination of the mandibular postural position through the simultaneous recording of electromagnetic articulography (EMA) and surface electromyography (sEMG). Two determination methods (no-command and swallow) are used to assess MPP in healthy patients and those with joint pathology, muscle pathology, and a combination of both, diagnosed with DC-TMDs. Additionally, the evaluation of mouth opening is conducted. Based on the EMA records, it is possible to describe the mouth opening using three different variables: vertical distance, Euclidean distance, and angle. The disposition of the sensors and the required mathematical operations are described. 

Since this research was conducted during the SARS-CoV-2 pandemic, the use of masks may have affected the masticatory muscles of the subjects [23].

## 2. Materials and Methods

### 2.1. Participants

A pilot study was conducted with a sample of sixteen participants (14 women, 2 men), recruited from the Temporomandibular Disorders and Orofacial Pain Polyclinic of the Universidad de La Frontera, Temuco, Chile. The sample selection process was conducted by non-probabilistic consecutive convenience sampling, where participants who met the inclusion and exclusion criteria were selected. Four groups were formed, differentiated by the diagnosis of a temporomandibular disorder: joint, muscular, mixed, and an asymptomatic group. The patient selection was performed by a dentist specialized in temporomandibular disorders and orofacial pain.

The diagnosis of temporomandibular disorders was conducted according to the Diagnostic Criteria for Temporomandibular Disorders (DC/TMDs), Spanish version, published in 2018 (https://buffalo.app.box.com/s/u9jd6bzk7sfai7o6wxwvsjchgtyu4zvh, accessed on 10 May 2023), by a dentist specializing in temporomandibular disorders and orofacial pain (C.C., dental surgeon), previously calibrated for diagnosis with DC/TMDs.

In accordance with the criteria described above, the participants were invited to be part of the study after signing an informed consent form approved by the Scientific Ethical Committee of the Universidad de La Frontera, Temuco, Chile (ACT N°087_18). 

#### Inclusion and Exclusion Criteria

The inclusion criteria covered patients aged 18 years or older, who visited the TMDs and Orofacial Pain Polyclinic of the Universidad de La Frontera due to TMDs.

Exclusion criteria covered patients with orofacial movement disorders, patients unable to follow instructions, patients with one or more missing teeth, patients with orthodontic appliances, and patients with oral lesions such as angular cheilitis and trauma affecting mandibular movement permanently, such as a mandibular fracture or injury occurring within the last year, that could explain or obscure the symptoms, as well as sequelae of cancer and lichen planus.

The study was conducted at the “Oral Physiology Laboratory” of the Research Center for Dental Sciences, Faculty of Dentistry, Universidad de La Frontera (Temuco, Chile).

### 2.2. Records

The movements were recorded simultaneously according to existing protocols for mouth opening movements [5] and vertical dimension [7], measured with a 3D electromagnetic articulograph (EMA) (AG501, Carstens Medizinelektronik, Bovenden, Germany) with a sampling frequency of 250 Hz, and the electromyographic activity was measured with a surface electromyograph (sEMG) (sEMG VIII, ArtOficio, Santiago, Chile) with a gain of 1000. 

#### 2.2.1. Equipment Assembly

**Figure 1:**
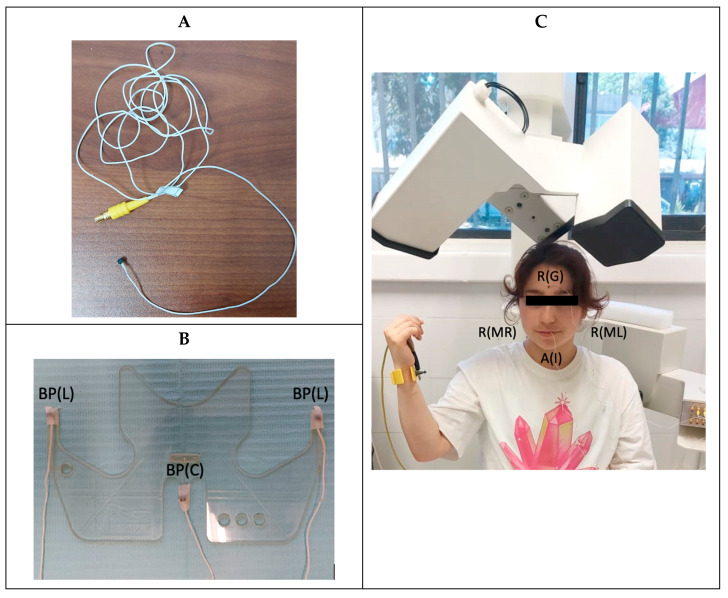
(**A**). Articulograph sensor. (**B**). Bite plane with sensors located in the central (BP(C)) and lateral areas (BP(L)) of the grooves of the accessory. (**C**). Reference sensors (R), positioned on the glabella (R(G)), right mastoid (R(MR)), and left mastoid (R(ML)). Active sensor (A), located in the interincisive line of the incisors of the mandible (A(I)).

Seven articulograph sensors were used, which were previously calibrated and numbered: three reference sensors, three sensors that were attached to the bite plane accessory, and one active sensor. These were connected according to the equipment connector number. The reference sensors were positioned on the glabella and the right and left mastoid. These were used to eliminate involuntary head movements so that they were not registered as mandibular movement. The bite plane is an accessory to the articulograph, which allows the origins of coordinates to be located in the occlusal plane. The bite plane sensors were placed in the central and lateral areas of the grooves of the accessory. The active sensor was placed in the interincisive line of the incisors of the mandible. In addition, the articulograph had a ground connector that was placed on the wrist of the patient (Figure 1).

**Figure 2:**
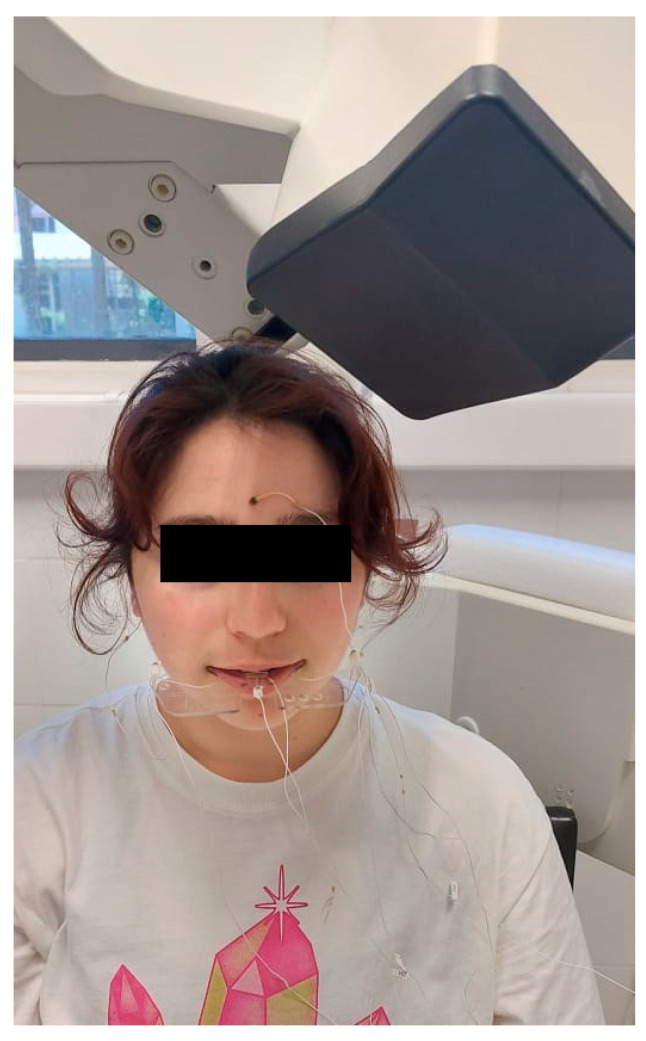
The patient is positioned under the EMA head with the reference sensors, active sensors, and bite plane in position.

The patient was instructed to remove any electronic and/or metallic items if present in order to avoid interference in the recordings. Once this was done, the participant was asked to sit under the EMA head in a comfortable position, upright and facing forward. The equipment sensors were then placed on the patient and the bite plane (Figure 2).

First, the bite plane sensors were attached with tape. Then, the skin was cleaned with alcohol, and the cutaneous, glabella, and mastoid sensors were glued with tissue adhesive (Epiglu^®^, Ober-Mörlen, Germany). To achieve better stability, part of the sensor cable was attached to the skin with tape. Finally, the active sensor was placed. The area where the active sensor was to be attached was dried with a paper napkin, and then the sensor was attached to the interincisive line with tissue adhesive. The grounding connector was placed on the wrist of each patient. Before it was placed, the skin was cleaned with alcohol and covered with a layer of conductive gel (Signa Gel, Parker Laboratories, Inc., Fairfield, NJ, USA, EE.UU.).

Afterwards, the sEMG sensors were placed. To do so, the patient was asked to squeeze and palpate the area, searching for muscle fibers. The sensors were attached to the patient using double-sided tape, provided by the sEMG equipment manufacturer, and conductive gel was placed over the electrodes. The electrodes were attached to the skin of the patient in such a way that the axis passing through both poles of the electrode was oriented parallel to the direction of the fibers. Four muscles were studied: the right and left masseter and right and left anterior temporal. The reference sensor was self-adhesive. As with the articulograph sensors, a part of the cable was glued to the skin for greater stability.

The sEMG (bandwidth 10–500 Hz with notch filter at 50 Hz) was configured with a gain of 1000. Four active sensors were used for bilateral acquisition of the masseter and temporalis, and a reference sensor located at the elbow.

#### 2.2.2. Baseline Records and EMA Active Sensor Placement

Before starting the recordings, a baseline record was made. The reference sensors and the bite plane sensors were assigned via the equipment interface. Once this was done, the participant was asked to put on the bite plane and hold it in his/her mouth (Figure 2). A 5-s recording was made, and the data were used to perform the head correction. This procedure is internal to the equipment and requires these 6 sensors. The reference sensors were used to eliminate head movements. The active sensor movement was relative to the reference sensors, so that if the patient moved his/her head, this movement was not registered. The bite plane sensors were used to transfer the origins of coordinates to the occlusal plane. The head correction was performed only once before starting the recordings. This enabled each subsequent record to eliminate the head movement and all of them to have the occlusal plane as a reference.

#### 2.2.3. Baseline Records and Placement of Active sEMG Sensors

The following tests were performed:

- Measurement at rest (MPP occurred);
- Maximum voluntary squeeze (the participant was asked to bite as hard as possible);
- Opening against resistance (the participant was asked to try to open the mouth while another person applied resistance against opening).

Once the participant was located under the EMA with all the EMA and sEMG sensors, both active and reference, and having performed both baseline recordings, the simultaneous EMA and sEMG records were obtained.

#### 2.2.4. DVO Protocol

For the DVO recording, the participant was instructed to reproduce the maximum intercuspidation position. To do so, the subject was instructed to keep his or her gaze straight ahead at a fixed point with voluntary, slight tooth contact and without any clenching force. This was done in order to record the initial point from which the rest of the mandibular movements would start.

#### 2.2.5. DVP Protocol

For the DVP record, the mandibular postural position was replicated using two methods.

(a) No-command method: The participant was instructed to maintain their gaze straight ahead at a fixed point, remaining comfortable and relaxed, with the teeth in inoclusion and lips in light contact.
(b) Swallowing method: The participant was asked to look straight ahead and swallow their saliva.

The physiological inclusion space value was used for DVP determination.

#### 2.2.6. Mouth Opening Protocol

For the recording of the different mouth openings, the participant was instructed to keep his or her eyes looking straight ahead at a fixed point and was asked to replicate the position of maximum mouth opening, independent of the presence of painful symptomatology; this opening was considered to be 100%.

Subsequently, a simple rule of 3 was used to calculate 75%, 50%, and 25% of the 100% value calculated in millimeters. This calculation was performed in order to reduce the sampling time.

Note: Each recording was made 1 time continuously. The subject in each recording held the position for 5 s.

### 2.3. Data Processing

The files resulting from each simultaneous EMA and sEMG record were processed by means of scripts developed in Matlab (MathWorks^®^, Natick, MA, USA). A script is a calculation routine formed by instructions, through which data are processed by applying mathematical operations, which enable the data to be processed, obtaining numerical parameters derived from the data and graphical representations, among others.

From the position EMA data, the interocclusal space measurement was performed using the 3D Euclidean distance (d) between the coordinates of each active sensor during MIC $\left(x_{1a},y_{1a},z_{1a}\right)$ and MPP $\left(x_{1b},y_{1b},z_{1b}\right)$ by applying Equation (1). To find the *z*-distance (vertical distance), the difference between the *z*-values of the MIC and MPP positions was simply calculated.

(1) $$d=\sqrt{{\left(x_{1a}-x_{1b}\right)}^{2}+{\left(y_{1a}-y_{1b}\right)}^{2}+{\left(z_{1a}-z_{1b}\right)}^{2}}$$

Using 3D vector algebra, the value of the mouth opening angle in MPP was obtained. For this purpose, the position data of the 3 sensors placed on the mandible were used; its coordinates are represented by the points $s_{1},s_{2}$, and $s_{3}$ in Equation (2). Equations (3) and (4) lead to the expression of a vector perpendicular to the plane passing through the points $s_{1},s_{2}$, and $s_{3}$. By Equation (6), the angle between these vectors was obtained, which was equivalent to the mouth opening angle.

(2) $$s_{1}=(x_{1},y_{1},z_{1});\;s_{2}=\left(x_{2},y_{2},z_{2}\right);\;s_{3}=(x_{3},y_{3},z_{3})$$

(3) $$v_{12}=s_{2}-s_{1}=(x_{2}-x_{1},y_{2}-y_{1},z_{2}-z_{1});\;v_{13}=s_{3}-s_{1}=(x_{3}-x_{1},y_{3}-y_{1},z_{3}-z_{1})$$

(4) $$v_{perpendicular}=v_{12}\times v_{13}$$

(5) $$Angle=cos^{-1}\left(\frac{{|v}_{perpendicular\_MIC}\cdot v_{perpendicular\_MPP}|}{\left|v_{perpendicular\_MIC}\right|\left|v_{perpendicular\_MPP}\right|}\right)$$

From the electromyography data, the root mean square of the sEMG signal (sEMGrms) was obtained (Equation (6)), applied over 50 ms windows without overlapping, and the mean of this signal was calculated, which was used as a representative parameter of the electromyographic activity acquired in each sEMG record.

(6) $$EMG_{rms}=\sqrt{\sum_{i=1}^{n}\frac{X_{i}^{2}}{n}}$$

where $X_{i}^{}$ is each of the elements of the sEMG signal.

### 2.4. Statistical Analysis

A descriptive data analysis was conducted using Excel to determine the mean and its respective standard deviation.

## 3. Results

Table 1 shows the results of the measurement of the physiological inoclusion space, determined by the mandibular postural position obtained through the no-command method and swallowing method, measured as the vertical distance, distance, and angle, and the electromyographic activity of the right and right masseters and left and right and left temporal, in healthy subjects and those with articular, muscular, and mixed pathologies. 

In relation to the vertical dimension, measured through the physiological inoclusion space, in the no-command group, regarding the MPP, the highest value of the Euclidean distance was obtained at 1.5 ± 0.7 mm (healthy group) and the lowest value at 0.7 ± 0.2 mm (articular group). In the group with the swallowing command to obtain the MPP, the greatest Euclidean distance was 2.5 ± 1.6 mm (muscular group), and the smallest was 1.4 ± 0.4 mm (healthy group) and 1.4 ± 0.3 mm (articular group). No great variability was observed between the groups. The distance (Euclidean distance) was described to be greater than the vertical distance (measured only on the z-axis) in all measurements, because it was the spatial distance between two points and there was a proportionality between them.

In relation to the angles, the highest value of 1.6° ± 1.7° was obtained in the no-command group and the lowest value of 0.3° ± 0.3° in the mixed and articular groups, respectively. In relation to the group with the swallowing command, the highest angle value was 3.9° ± 6.5° and the lowest value was 0.4° ± 0.5°, in the mixed and articular groups, respectively.

Electromyographic activity in the no-command group for MPP determination was 7.8 ± 3.4 μV in the group with muscle TMDs in the left temporalis muscle, with the highest value, and 4.1 ± 0.4 μV measured in the right temporalis of the mixed group as the lowest value. In the group with a command, the highest value was 8.8 ± 7.1 μV for the left temporalis muscle of the healthy group, and the lowest value was 4.3 ± 0.7 μV for the right temporalis muscle of the mixed group.

Table 2 shows the results of the measurement of the oral opening at 100%, 75%, 50%, and 25%, measured as the vertical distance, distance, and angle in healthy subjects with articular, muscular, and mixed pathologies. The electromyographic activity of the right and left masseter and right and left temporal bone was recorded.

The values of the maximum openings measured on the z-axis indicated as 100% were between 45.2 ± 3.8 mm (healthy group) and 36.9 ± 4.9 mm (mixed group). The openings indicated as 75% obtained values between 33.5 ± 3.1 mm (healthy group) and 26.3 ± 4.4 mm (mixed group). The openings indicated as 50% resulted in values between 22.4 ± 4 mm and 18 ± 3.2 mm (muscular and mixed groups, respectively). The openings indicated as 25% presented values between 11.3 ± 2 mm and 8.9 ± 1.6 mm (muscular and articular groups, respectively). 

The Euclidean distance for the 100% openings described values between 50.1 ± 4.1 mm and 38.3 ± 5.1 mm (healthy and mixed groups, respectively) for the 75% group, and it was between 34.9 ± 2.9 mm and 27.2 ± 3.6 mm for the healthy and mixed groups, respectively. For the 50% opening group, the distances were between 23.7 ± 3.2 mm and 18.5 ± 2.9 mm for the muscular and mixed groups, respectively. Finally, for the opening at 25%, the distance was 11.9 ± 2 mm for the muscle group and 9.6 ± 1.4 mm for the articular group.

Maximum opening angles (100%) were recorded between 33.6° ± 5.3° (healthy group) and 29.2° ± 9.1° (articular group). Mouth opening angles at 75% were recorded between 22.5° ± 10.7° (mixed group) and 18.3° ± 3° (muscular group). Mouth opening angles at 50% were recorded between 16.6° ± 11.6° (mixed group) and 11.4° ± 2.2° (muscular group). Finally, mouth opening angles at 25% were recorded between 11.0° ± 10.4° (mixed group) and 6° ± 0.7° (healthy group). The measurement of the angles evidenced high variability in the mixed group for all the opening levels.

In the openings at 100%, the highest electromyographic activity recorded was 20.3 ± 1.2 μV (left temporalis muscle) in the muscle group, and the lowest was 6.5 ± 1.9 μV (right temporalis) in the healthy group. For the 75% openings, the highest electromyographic activity was seen in the muscle group with 9 ± 1.9 μV (left temporalis), and the lowest activity was seen in the articular group with 5.4 ± 0.2 μV (left temporalis). For the openings at 50%, greater electromyographic activity was obtained in the muscle group with 7.5 ± 3 μV (left temporal) and the lowest electromyographic activity was 5.15 ± 1.5 μV in the mixed group (right temporal). For the opening of 25%, patients presented higher electromyographic activity of 7.6 ± 3 μV (left temporal) in the muscle group, and the lowest electromyographic activity was noted in the group of healthy patients at 4.8 ± 1.5 μV (left temporal). 

## 4. Discussion

In this study, the physiological inocclusion space was evaluated from the MPP and the mouth opening movement in healthy patients and those with TMDs as diagnosed by DC/TMDs [18], i.e., with articular, muscular, and mixed pathologies, measured simultaneously with EMA and sEMG, obtaining an accurate measurement of the Euclidean distance and the distance in the z-axis, in contrast to other measurement methods [14,24,25,26,27]. An angular measurement of the oral opening was also performed with the data obtained.

The physiological inoculation space was obtained from the determination of the MPP through the measurement methods with a swallowing command and no command. The results indicated that both were simple to use, and the values obtained were within the ranges described by other authors [28,29], i.e., between 1 and 3 mm, and slightly lower than those obtained by authors who described ranges between 2 and 4 mm. The authors of [30,31,32,33] measured the variables with less accurate instruments.

The highest EIF obtained with the no-command technique was 1.5 ± 0.7 mm, 1.3 ± 1.2 mm, 1.1 ± 1.2 mm, and 0.7 ± 0.2 mm in the healthy, mixed, muscular, and articular patient groups, respectively. The EIF values obtained with the swallowing technique were 2.5 ± 1.6 mm, 1.7 ± 1.5 mm, 1.4 ± 0.4 mm, and 1.4 ± 0.3 mm in the muscular, mixed, healthy, and articular groups, respectively. It is evident that the groups with pathologies presented variability at the time of performing commands such as swallowing, modifying the MPP, probably due to the muscular response, in contrast to the healthy patients, who maintained an EIF close to 1.5, measured with no command. This could indicate the importance of providing treatment in these groups prior to oral rehabilitation, which requires the modification, return, or creation of a new vertical dimension.

The highest electromyographic activity recorded was 8.8 ± 7.1 μV in the left temporalis muscle using the swallowing technique in healthy patients. The lowest value was 4.11 ± 0.43 μV in the right temporalis muscle in patients with a mixed pathology and using the no-command technique, thus not showing great variability between groups. This differs from the work by Gomez et al. [34], which described asymmetry between the left and right sides in healthy patients.

The range of oral opening was individualized through percentages, differing from what has been described [34], where the openings were measured every 3 mm, in order to reduce the measurement factors that could interfere with the results; a smaller number of measurements was favored by means of individualization, with percentages of 25%, 50%, and 75% calculated on the basis of the 100% maximum opening of each participant, proving to be a very good alternative in patients with pathology, allowing individualization according to the mouth opening range of each participant. The values of the Euclidean distance at 100%, 75%, 50%, and 25% were always greater than the distance on the *z*-axis, which was consistent.

The measurement of the angles in the openings showed a tendency toward high variability in the group of participants with a mixed pathology in all the measurements, 100% 75%, 50%, and 25%, different to that observed in the other groups. This could be associated with an alteration in the articular and muscular proprioception, which, being concomitant in patients with mixed pathology, can cause the modification of the efferent alpha-gamma response of the stomatognathic system.

The electromyographic activity in the mouth openings at the different percentages ranged from 20.3 ± 1.2 μV to 6.5 ± 1.9 μV for the maximum opening group, and values between 7.63 ± 3.02 and 4.80 ± 1.50, with a slight increase in electromyographic activity, were recorded as the mouth opening percentages increased.

This pilot study had the limitation of a small number of participants in each group. Therefore, no meaningful comparisons could be made between groups.

Recent studies have revealed a relationship between patients with visual impairments (myopia and hyperopia) and the muscular activity of the stomatognathic system [35,36]. This was another limitation of the present study, since the visual impairments of patients were not examined, and this could have influenced the obtained results.

## 5. Conclusions

The analysis of sEMG activity in different mandibular positions recorded by EMA (MPP, MIC, and different mouth opening degrees) for healthy subjects and patients with TMDs is possible. Determination of EIF was conducted in a simple manner. Compared with previous studies, this was the first case where the vertical distance, Euclidean distance, and angle were simultaneously evaluated without the need for devices attached to the head. The introduction of the angle and normalization of mouth opening can provide a new tool to evaluate sEMG activity in relation to mouth opening, since mouth opening is influenced by the subject’s dimension and the sEMG signals cannot be directly compared. Electromagnetic articulography unifies the recording of the mouth position and adds a 3D analysis. EMA shows limitations in research that involves metal implants as metal interferes with electromagnetic fields and leads to incorrect measurements.

## Figures and Tables

**Table 1:** MPP EMA and sEMG analysis of the four groups with no-command and swallowing procedures. In EMA vertical, Euclidean distance and angle are described. sEMG analysis shows the mean activity of sEMGrms for each muscle.

		EMA			sEMGrms			
	MPP	Vertical Distance [mm]	Distance [mm]	Angle [°]	MR [μV]	ML [μV]	TR [μV]	TL [μV]
Healthy N = 5	No Command	1.2 ± 0.79	1.5 ± 0.78	0.7 ± 0.41	5.51 ± 1.09	5.36 ± 0.89	5.25 ± 0.94	4.66 ± 1.30
	Swallowing	0.9 ± 0.79	1.4 ± 0.42	0.6 ± 0.16	7.04 ± 2.03	6.72 ± 2.79	7.58 ± 4.54	8.81 ± 7.19
Joint N = 3	No Command	0.6 ± 0.13	0.7 ± 0.23	0.3 ± 0.37	7.17 ± 2.18	6.17 ± 1.94	7.02 ± 3.21	6.61 ± 1.92
	Swallowing	1.1 ± 0.55	1.4 ± 0.32	0.4 ± 0.50	6.95 ± 2.12	5.78 ± 1.58	6.47 ± 2.31	5.86 ± 1.12
Muscular N = 3	No Command	0.9 ± 1.21	1.1 ± 1.22	0.9 ± 0.74	7.58 ± 0.26	7.20 ± 1.52	6.62 ± 1.50	7.89 ± 3.45
	Swallowing	2.2 ± 1.45	2.5 ± 1.61	1.4 ± 0.96	5.90 ± 2.26	6.58 ± 1.89	5.53 ± 0.53	7.09 ± 2.30
Mixed N = 5	No Command	1.2 ± 1.13	1.3 ± 1.21	1.6 ± 1.78	5.64 ± 2.45	5.70 ± 2.38	4.11 ± 0.43	6.78 ± 3.99
	Swallowing	1.5 ± 1.45	1.7 ± 1.56	3.9 ± 6.65	5.66 ± 2.64	6.03 ± 2.58	4.37 ± 0.75	6.84 ± 3.85

MR = right masseter muscle, ML = left masseter muscle, TR = right temporal muscle, and TL = left temporal muscle.

**Table 2:** EMA and sEMG analysis of the four groups for 25%, 50%, 75%, and 100% of mouth opening. In EMA vertical, Euclidean distance and angle are described. sEMG analysis shows the mean activity of sEMGrms for each muscle.

		EMA			sEMGrms			
	Oral Opening	Vertical Distance [mm]	Distance [mm]	Angle [°]	MR [μV]	ML [μV]	TR [μV]	TL [μV]
Healthy	100%	45.2 ± 3.83	50.1 ± 4.10	33.6 ± 5.35	7.04 ± 0.83	6.75 ± 1.99	6.55 ± 1.92	7.22 ± 3.75
Articular		38.7 ± 6.94	43.1 ± 7.38	29.2 ± 9.13	11.93 ± 7.66	13.68 ± 10.47	6.04 ± 1.14	9.54 ± 2.95
Muscular		44.5 ± 9.04	48.5 ± 10.68	33.0 ± 7.82	8.20 ± 3.46	8.71 ± 4.15	10.47 ± 6.74	20.39 ± 1.20
Mixed		36.9 ± 4.99	38.3 ± 5.19	30.1 ± 9.52	6.12 ± 3.17	7.78 ± 4.44	7.64 ± 3.31	10.74 ± 5.75
Healthy	75%	33.5 ± 3.19	34.9 ± 2.93	20.9 ± 3.04	6.58 ± 2.06	6.98 ± 4.11	5.97 ± 1.59	6.69 ± 1.83
Articular		28.2 ± 4.09	29.6 ± 3.24	18.4 ± 3.68	6.77 ± 1.67	5.89 ± 1.23	5.42 ± 0.29	5.67 ± 0.60
Muscular		28.0 ± 1.50	29.8 ± 2.09	18.3 ± 3.02	5.89 ± 1.90	6.49 ± 2.08	6.32 ± 1.21	9.00 ± 1.90
Mixed		26.3 ± 4.44	27.2 ± 3.66	22.5 ± 10.77	6.24 ± 1.38	5.73 ± 1.41	6.01 ± 1.95	7.70 ± 3.70
Healthy	50%	21.9 ± 1.74	23.2 ± 1.32	12.7 ± 1.57	6.09 ± 0.80	5.85 ± 1.53	5.29 ± 1.03	5.30 ± 1.76
Articular		19.0 ± 2.77	19.8 ± 2.78	11.4 ± 2.29	6.82 ± 2.20	5.54 ± 1.45	5.54 ± 0.42	5.99 ± 0.66
Muscular		22.4 ± 4.01	23.7 ± 3.26	14.1 ± 0.71	5.89 ± 2.26	6.22 ± 1.89	6.13 ± 0.97	7.51 ± 3.00
Mixed		18.0 ± 3.20	18.5 ± 2.99	16.6 ± 11.65	5.44 ± 2.36	5.23 ± 1.85	5.15 ± 1.58	7.56 ± 3.88
Healthy	25%	10.6 ± 0.75	11.3 ± 1.15	6.0 ± 0.76	6.09 ± 1.04	5.74 ± 1.44	5.16 ± 1.31	4.80 ± 1.50
Articular		8.9 ± 1.63	9.6 ± 1.44	5.9 ± 1.02	6.83 ± 2.06	5.41 ± 1.33	5.70 ± 0.65	6.09 ± 0.61
Muscular		11.3 ± 2.05	11.9 ± 2.08	7.0 ± 0.60	5.77 ± 2.21	6.99 ± 2.78	6.02 ± 0.67	7.63 ± 3.02
Mixed		9.7 ± 1.90	10.1 ± 1.72	11.0 ± 10.49	5.38 ± 2.35	5.56 ± 2.62	5.27 ± 2.32	6.99 ± 3.68

MR = right masseter muscle, ML = left masseter muscle, TR = right temporal muscle, and TL = left temporal muscle.

## Data Availability

Not applicable.
